# Altered Serum Proteins Suggest Inflammation, Fibrogenesis and Angiogenesis in Adult Patients with a Fontan Circulation

**DOI:** 10.3390/ijms25105416

**Published:** 2024-05-16

**Authors:** Miriam Michel, David Renaud, Ronny Schmidt, Matthias Einkemmer, Lea Valesca Laser, Erik Michel, Karl Otto Dubowy, Daniela Karall, Kai Thorsten Laser, Sabine Scholl-Bürgi

**Affiliations:** 1 Department of Child and Adolescent Health, Division of Pediatrics III—Cardiology, Pulmonology, Allergology and Cystic Fibrosis, Medical University of Innsbruck, 6020 Innsbruck, Austria; matthias.einkemmer@student.i-med.ac.at; 2 Fundamental and Biomedical Sciences, Paris-Cité University, 75006 Paris, France; david.renaud.chd@gmail.com; 3 Health Sciences Faculty, Universidad Europea Miguel de Cervantes, 47012 Valladolid, Spain; 4 Sciomics GmbH, 69151 Neckargemünd, Germany; ronny.schmidt@sciomics.de; 5 Center of Pediatric Cardiology and Congenital Heart Disease, Heart and Diabetes Center North Rhine-Westphalia, Ruhr-University of Bochum, 32545 Bad Oeynhausen, Germany; leavalescalaser@gmail.com (L.V.L.); kodubowy@hdz-nrw.de (K.O.D.); tlaser@hdz-nrw.de (K.T.L.); 6 Clinic for Pediatrics, Medizin Campus Bodensee, 88048 Friedrichshafen, Germany; erik.michel@web.de; 7 Department of Child and Adolescent Health, Division Pediatrics I—Inherited Metabolic Disorders, Medical University of Innsbruck, 6020 Innsbruck, Austriasabine.scholl-buergi@tirol-kliniken.at (S.S.-B.)

**Keywords:** angiogenesis, fibrogenesis, Fontan, glycophorin-A, inflammation, leukemia inhibitory factor, nerve growth factor-ß, proteomics, signaling, syndecan-1

## Abstract

Previous omics research in patients with complex congenital heart disease and single-ventricle circulation (irrespective of the stage of palliative repair) revealed alterations in cardiac and systemic metabolism, inter alia abnormalities in energy metabolism, and inflammation, oxidative stress or endothelial dysfunction. We employed an affinity-proteomics approach focused on cell surface markers, cytokines, and chemokines in the serum of 20 adult Fontan patients with a good functioning systemic left ventricle, and we 20 matched controls to reveal any specific processes on a cellular level. Analysis of 349 proteins revealed 4 altered protein levels related to chronic inflammation, with elevated levels of syndecan-1 and glycophorin-A, as well as decreased levels of leukemia inhibitory factor and nerve growth factor-ß in Fontan patients compared to controls. All in all, this means that Fontan circulation carries specific physiological and metabolic instabilities, including chronic inflammation, oxidative stress imbalance, and consequently, possible damage to cell structure and alterations in translational pathways. A combination of proteomics-based biomarkers and the traditional biomarkers (uric acid, γGT, and cholesterol) performed best in classification (patient vs. control). A metabolism- and signaling-based approach may be helpful for a better understanding of Fontan (patho-)physiology. Syndecan-1, glycophorin-A, leukemia inhibitory factor, and nerve growth factor-ß, especially in combination with uric acid, γGT, and cholesterol, might be interesting candidate parameters to complement traditional diagnostic imaging tools and the determination of traditional biomarkers, yielding a better understanding of the development of comorbidities in Fontan patients, and they may play a future role in the identification of targets to mitigate inflammation and comorbidities in Fontan patients.

## 1. Introduction

The Fontan operation is the final set of palliative surgical procedures for children born with single-ventricle (SV) heart disease. While several forms exist [1] and the debate over the most appropriate form is still current [2], it is generally realized through the anastomosis of the vena cava on the pulmonary artery, allowing the blood to go passively into the lungs and the single ventricle supplying the systemic circulation. The criteria for the competition of total cavopulmonary connection has evolved over time [3], and the majority of patients with univentricular heart diseases are now expected to reach the Fontan stage [4]. It allows for separation of the pulmonary and systemic circulation and a substantial improvement in arterial oxygen saturation [5]. This anatomic setup results in the lack of a subpulmonary ventricular pump and passive non-pulsatile pulmonary blood flow from the superior and inferior vena cava, leading to elevated resting central venous pressure (CVP) and venous congestion [1,6]. It is estimated that 70,000 patients live with this univentricular circulation. It is expected that 80% of them will reach an age of 30 years free of transplantation [7], though not all patients perform equally, and Fontan patients with an underlying morphologically right ventricle currently exhibit a much poorer prognosis [8]. While surgical outcomes are now excellent, with several cohorts showing no perioperative death [9,10], long-term outcomes are still uncertain. A Fontan circulation might come with multiple complications and comorbidities, including heart failure (HF), arrhythmia, atrioventricular valve regurgitation, thromboembolic disease, Fontan-associated liver disease, protein-losing enteropathy, plastic bronchitis, renal disease, and restrictive lung disease [1,6,11,12].

As procedural techniques improve and the population of Fontan patients grows older, the focus is shifting to the prevention and management of Fontan complications with the goal of maximizing quality of life and circulatory functional capacity. To facilitate the appropriate care of these patients, dedicated Fontan surveillance programs are used to monitor the development of subclinical complications [13,14,15]. However, detection, the indication and timing of intervention, and the outcome prediction of comorbidities and their evaluation in terms of Fontan circulation as their origin—or, vice versa, their impact on the Fontan circulation—are challenging.

The assessment of the metabolism of Fontan patients is a recent attempt to detect subtle alterations of the Fontan cardiac, circulatory, and systemic system [16,17,18,19]. Recently, comparative omics studies have been published, showing metabolic perturbations hinting at derangements in energy metabolism and inflammation, oxidative stress, endothelial dysfunction, and elevated myocardial protein turnover [20]. Many of these findings in Fontan patients reflect changes reported in biventricular (BV) patients with congestive HF [21,22,23].

Omics studies in SV patients of different ages, and thus across all steps toward Fontan palliation, suggest that even in the youngest children (interstage—after Norwood operation and before superior vena cava connection to the pulmonary artery, and patients with various morphological cardiac subtypes) [24,25,26], abnormalities in cardiac and systemic metabolism are present. Particularly impaired function of the mitochondria has been shown, indicating that, irrespective of the actual palliation stage, the SV (and even the clinically non-failing SV) circulation represents a fragile equilibrium stage, rendering the SV prone to failure.

As for systemic alterations, alterations across most organ systems were found. Apart from energy metabolism, the alterations were mostly related to inflammation, angiogenesis and fibrogenesis, and immune system activation, which can be assigned mostly to the gastrointestinal system, neural and cardiac physiology, and development [25].

Based on these pioneering works, our objective was to further investigate serum proteomics in a cohort of adult Fontan patients (good exercise capacity, systemic left ventricle (LV)), to reveal any specific processes on a cellular level. Using a broad immune-based antibody array (‘affinity-proteomics’) approach, we focused on serum protein patterns, among them cell surface markers, cytokines, and chemokines, to more precisely characterize especially regulatory processes in this ‘good’ patient cohort.

## 2. Results

### 2.1. Clinical Examination, Routine Laboratory Analytes, Imaging, Exercise Capacity Testing

After applying all inclusion and exclusion criteria, 20 adult Fontan patients with a good functioning systemic LV without more than mild atrioventricular valve regurgitation were selected for the study. The results of “traditional” examinations (patient and control clinical assessment, exercise capacity testing, routine laboratory analyses) are set out in our recent works on lipid and amino acids metabolism (Table 1) [21,22].

### 2.2. Proteomics Analysis

The scioCD assay covers 349 unique protein targets. Of those unique protein targets, 119 are cytokines/chemokines and 141 are cell surface markers. The remaining 89 protein targets are integrins, kinases, antibody chains, and others. For detection, 526 antibodies were used (some cell surface targets were detected by more than one antibody) (Supplementary Table S1).

#### 2.2.1. Differentially Abundant Proteins between Patients and Controls

**Figure 1:**
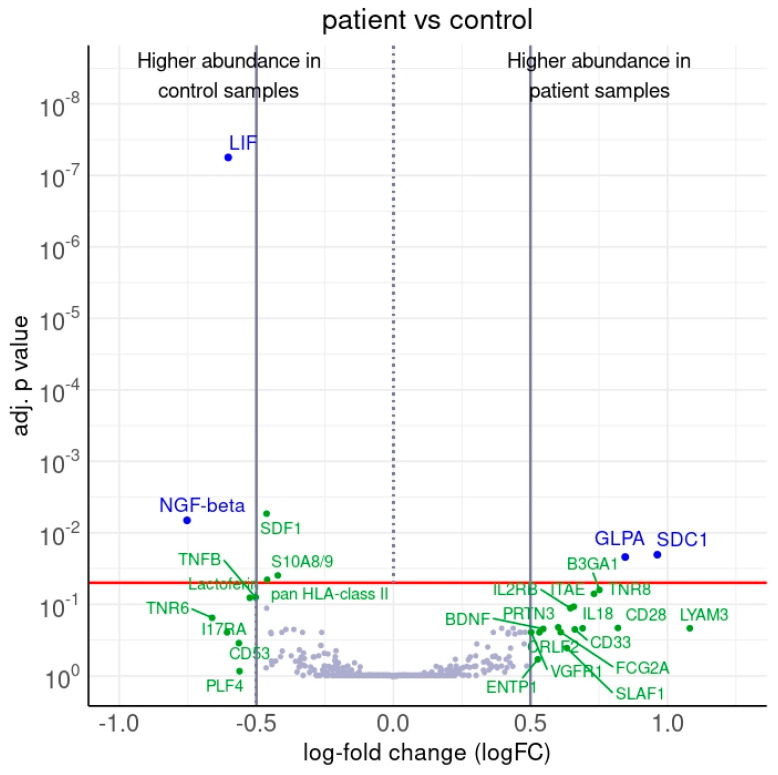
Distinct abundance variations of proteins in patient and control serum samples (volcano plot). The plot visualizes the adjusted *p*-values and corresponding log-fold changes (|logFC|). *p* < 0.05 was considered statistically significant (horizontal red line). The |logFC| cutoffs are indicated as vertical lines. Proteins with a positive |logFC| had a higher abundance in patient samples; proteins with a negative value were in control samples. Proteins with |logFC| < 0.5 and a significant adjusted *p*-value are defined as differential and are displayed in blue. Proteins indicated in green either feature a |logFC| > 0.5, while not reaching the significance threshold, or feature a significant difference, while not reaching the |logFC| threshold.

**Figure 2:**
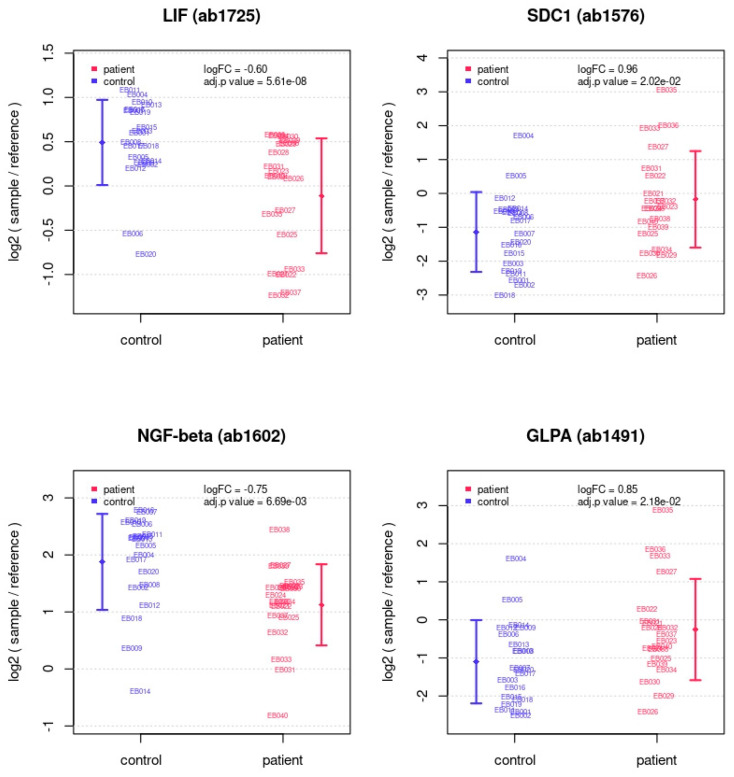
Individual array values for the four proteins with differential abundance in patient and control samples (see Table 2). Each sample is measured by four replicate spots per array. Rhombs indicate sample group means. Whiskers indicate one standard deviation.

**Figure 3:**
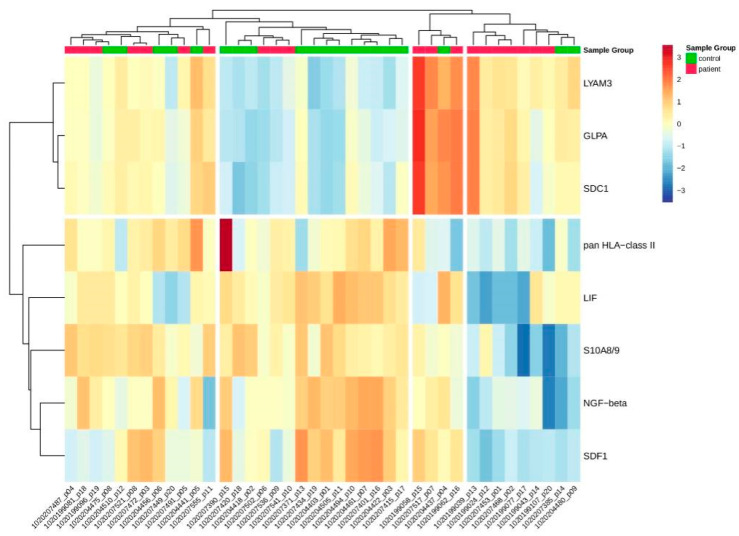
Heatmap displaying the relative expression of proteins identified as differential. Values were centered and scaled by proteins.

Between patient and control samples, four antibodies recorded a differential protein abundance (adj. *p*-value < 0.05, |logFC| > 0.5). The proteome of the patients showed an increased abundance of syndecan 1 (SDC1) and glycophorin-A (GLPA), and a decrease in leukemia inhibiting factor (LIF) and nerve growth factor-β (NGF-β) (Figure 1 and Figure 2, Table 2). Further proteins exhibited a differential abundance in patient and control samples with either a notable or significant |logFC| (Table 3, Figure 3).

#### 2.2.2. Principal Component Analysis

**Figure 4:**
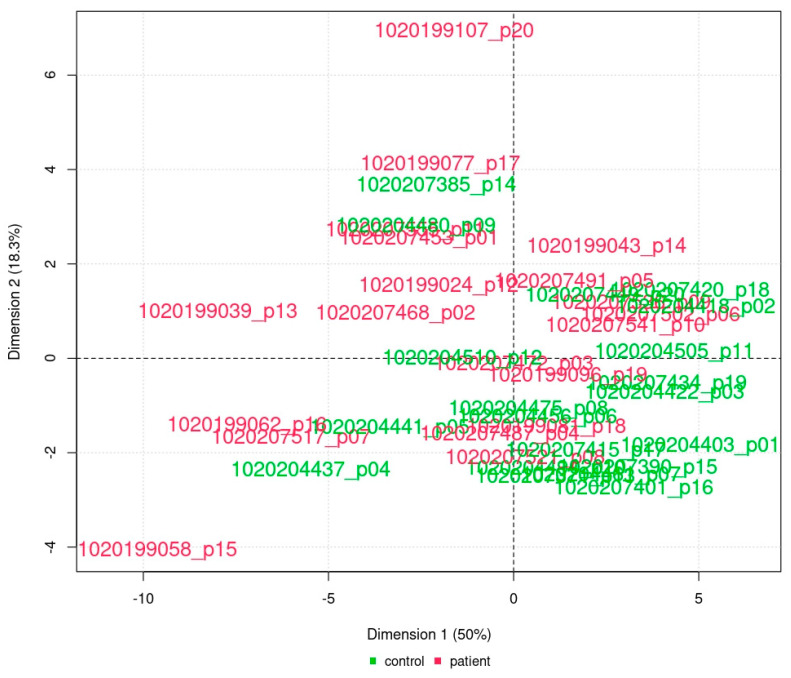
Principal component analysis for differential proteins. The scatter plot displays the first two principal components of the samples’ protein signal data based exclusively on the four differentially abundant proteins GLPA, LIF, SDC1, and NGF-β. In the plot, the location of the samples is defined by their first two principal components, i.e., linear combinations of protein features with the largest variance across the samples. Samples with a similar profile are located in close proximity. The percentages given in the axis labels describe the ratio of total variance explained by the respective principal component. Note that in the principal component analysis of the four differential proteins, the distribution of the probands suggests a clustering of patients or controls. Green, controls; red, patients.

A principal component analysis was performed for the array data and the array data filtered for differential proteins. Only the analysis performed for the four differential proteins implies a clustering of controls vs. patients (Figure 4).

#### 2.2.3. Receiver Operating Characteristic (ROC) Analysis

**Figure 5:**
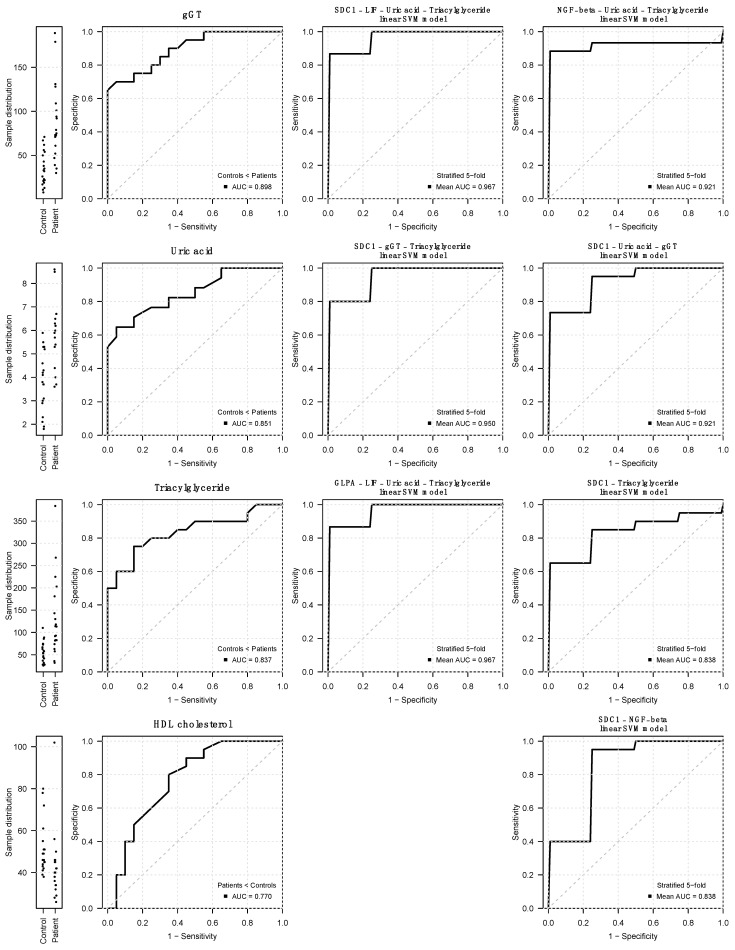
ROC curves for selected parameters. Receiver operating characteristic curves and area under curves for selected traditional and proteomics serum parameters and for combinations thereof with regard to group assignment (patient vs. control). Note the exceeding performance of combinations including γGT, uric acid, or triglyceride serum concentration in combination with one or two of the proteomics variables. The dashed line represents an area under the curve of 0.5.

While the accuracy in discriminating between patients and controls was acceptable to good for traditional laboratory parameters considered individually (gGT AUC 0.898; uric acid AUC 0.851; triacylglycerides AUC 0.837; HDL AUC 0.77), in combination with <2 analytes, the discrimination performance yielded excellent (AUC > 0.8) results (combination of SDC1, LIF, uric acid, and triacylglycerides AUC 0.967; combination of GLPA, LIF, uric acid, and triacylglycerides AUC 0.967; combination of SDC1, gGT, and triacylglycerides AUC 0.95; combination of NGF-β, uric acid, and triacylglycerides AUC 0.921; combination of SDC1, uric acid, and triacylglycerides AUC 0.921; combination of SDC1 and NGF-β AUC 0.838; combination of SDC1 and uric acid AUC 0.838) (Figure 5).

#### 2.2.4. Random Forest Analysis of Proteomics Parameters with Respect to Group Assignment

**Figure 6:**
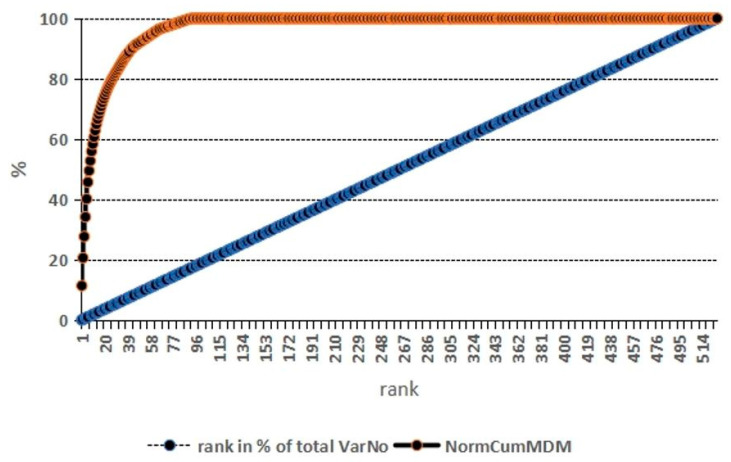
Normalized cumulated MDM (proteomics). Cumulative impact of the 526 proteomics-derived analytes examined on classification competence into diseased vs. non-diseased proband serum (dotted curve, red). The linear line (blue) represents the analytes’ rank standardized to 100%. Open circle: the 27/526 topmost ranked proteins (5% of analytes) account for 80% of classification optimum. MDM, mean decrease in the margin (classification impact score).

We found a good agreement between the rankings by RF or classical statistics among the topmost-ranked analytes (Table 4, Figure 6). After conducting repeated RF runs, all results proved robust with regard to variations in the RF parameter setting. Each run took only a few seconds to complete.

## 3. Discussion

By using a targeted proteomics approach, we sought to get detailed insight into the pathophysiology of Fontan patients as a first step toward the identification of new diagnostic and therapeutic targets.

### 3.1. Fontan Is Never Normal—‘Good’ Fontan Patients Exhibit a Specific Proteomics Phenotype

The primary finding of our study is that the ‘good’ Fontan patients with a systemic LV exhibit a different serum proteomic pattern compared to their matched control. Neither ‘general baseline factors’ like the proband‘s age and sex, nor factors with potential bias such as the accuracy of pre-analysis, had a major influence on the results. Our findings are in line with the specific physiology of the Fontan. The left and right ventricles differ in structure as well as in the transcriptional pathways involved [29]. The ‘good’ Fontan heart exhibits a specific metabolic phenotype [21,22] with an increased tendency toward instability [24]. Traditional markers for HF such as brain natriuretic peptide (BNP) or N-terminal-pro-brain natriuretic peptide (NT-pro-BNP) find their limits in understanding Fontan circulation [30]. Omics might be able to detect subtle levels of HF below the sensitivity of traditional markers for HF [22]. Current efforts are being directed toward analyzing the potential of innovative biomarkers in Fontan patients, such as growth/differentiation factor-15 [31] or cancer antigen-125 [32], to better understand their prognosis and long-term health outcomes [33].

### 3.2. Four Proteins as Inflammatory Biomarkers at Different Levels in Fontan Patients

The next important finding of our study is that we detected four distinct proteins with serum levels different in Fontan patients compared to matched controls, namely SCD1, GLPA, LIF, and NGF-β. In keeping with the metabolomics results of alterations of serum levels of phospholipids and derivations of the nitric oxide-metabolism in the same patient group [21,22], these four analytes have been described to be involved in inflammatory pathways as part of the regulation of myocardial stress and protection [34,35,36,37]. Inflammation is the natural response of the body to fend off injury or infection. It is recognized to play a critical role in the etiology of cardiovascular diseases [38] as well as HF [39]. Essential steps are both related to inflammation (recognition of the stimulus, often through PPARs; release of pro-inflammatory molecules such as cytokines; recruitment of the immune cells) and the resolution thereof [40]. Recent research has discussed immunity and inflammation as key ignored players in the treatment of congenital heart disease [41]. The significance of thymectomy in the repair of congenital heart diseases is questioned, specifically concerning its impact on immunity and cytokine alterations [42]. From a clinical perspective, elevated SDC1 could relate to inflammation, cardiac and/or hepatic fibrosis, and endothelial damage. While traditionally connected with mutagenicity, elevated GLPA could relate to alteration of red blood cell structure observed in Glenn and Fontan patients [43]. Decreased LIF could relate to a fragile heart on the metabolic level, rendering it less capable of coping with mechanical or metabolic stress. Decreased NGF-β could relate to an acquired alteration of the autonomic nervous system, found to be a consequence of Fontan completion and worsening over time [44,45]. Of note is that traditional parameters for inflammation (C-reactive protein, leucocyte count) or HR (NT-pro BNP) were normal in our patients. Even more intriguing is that other standard biomarkers for inflammation like tumor necrosis factor-α, interleukin-1, or interleukin-12, which are part of the analytes determined by our proteomics approach, were not different between patients and controls. Does that mean that subtle alterations of SCD1, GLPA, LIF, and NGF-β herald any changes in the classical markers of inflammation?

#### 3.2.1. Elevated SDC1—Inflammation, Cardiac and Hepatic Fibrosis, Angiogenesis

SDC1 is a transmembrane heparan sulfate proteoglycan expressed in many organs, including the vascular system, heart, and liver [46]. It connects the surface cells to extracellular matrix components such as collagens, fibronectins, and thrombospondin. In that way, it plays a crucial role in cell adhesion, cell signaling, the extracellular matrix, and the regulation of cellular processes through interaction with a wide number of proteins such as TGF-beta, heparin-binding EGF-like growth factor, PDGF, VEGFs, and FGFs. The protein consists of a core protein anchored to the cell membrane, with long chains of heparan sulfate glycosaminoglycans attached to it. SDC1 can become a soluble heparan sulfate proteoglycan. SDC1 shedding is seen as a post-translational mechanism related to several inflammatory and pathophysiological diseases, with both pro-inflammatory and anti-inflammatory properties [34]. Many studies have highlighted its role in the development of cardiac fibrosis, angiogenesis, or atherogenesis; it is associated with the activation of angiotensin II, specifically regulating inflammatory and regenerative responses [47,48]. In the heart, the SDC1 level correlates with fibrosis and inflammation in patients with HF and non-ischemic dilated cardiomyopathy [49]. SDC1 can worsen cardiac fibrosis induced by angiotensin II through the angiotensin/TGF-beta pathway [50]. In the liver, SDC1 levels are the highest in liver cirrhosis and hepatocellular carcinoma. While initially being protective in the early stages of liver fibrosis, this is a short-term effect [51]. In the vascular system, SDC1 is the main component of the endothelial glycocalyx [52]. Upon glycocalyx degradation, heparan sulfate chains are released into the serum as a post-translational signal. SDC1 has immunomodulatory properties [53], modulating motility and resolution responses of macrophages relevant to plaque destabilization in atherosclerosis [54]. High serum levels of SDC1 were observed in the acute perioperative period after the repair of congenital heart defects [55]. Under those various inflammatory conditions, shedded SDC1 binds with different growth factors and initially inhibits fibrosis. This mechanism is of only short duration, ending in a significant decrease in SDC1 and loss of heparan sulfate [47]. High SDC1 serum levels are generally seen as a marker of poor prognosis. In the literature, there is a debate about whether SDC1 is a marker of early inflammation or of disease severity [34]. In our study, SDC1 was elevated in ‘good’ adult Fontan patients compared to matched controls—it would be highly interesting to understand patients’ extent of collateral vessels in the Fontan circuit and their general hepatic status: patients with collateral vessels may have lower central venous pressures and may (to a certain degree) have preserved Fontan hemodynamics; this may prevent the development of or limit the extent of liver disease. Our patients did not exhibit laboratory signs of impaired hepatic synthetic function or manifest hepatic injury. However, they exhibited signs of cholestasis (frequently seen in Fontan patients). Of note, serum SDC1 levels are dependent on kidney function [56]. From our data, we cannot determine the source of SDC1.

#### 3.2.2. Elevated GLPA—Inflammation, Mutagenicity, Red Blood Cell Structure

GLPA is a membrane glycoprotein primarily found on the surface of red blood cells, where it is the most abundant. The carbohydrate chains of GLPA play a role in cell recognition and interaction with pathogens, such as bacteria and viruses, and blood group antigens [57,58]. Glycophorins might decrease signals to natural killer cells [59]. Currently, a common clinical use of GLPA is its application in mutagenicity assays after exposition to ionizing radiation [60] or chemicals [61], as it is thought to be indicative of a higher-than-normal amount of DNA damage. One of the known functions of GLPA is its involvement in the maintenance of the erythrocyte’s characteristic biconcave shape and resistance to deformation under shear stress. Fontan patients are known to have a specific rheologic phenotype. Their blood viscosity is lowered due to decreased red cell aggregation and higher deformability [62]. The ligation of GLPA generates reactive oxidative species, leading to decreased red blood cell function and inflammation [35]. Paradoxically, our findings are an elevation of GLPA protein, which so far we cannot interpret considering the Fontan rheology: increased GLPA would mean increased rigidity, while rheological studies on SV found decreased rigidity [43].

#### 3.2.3. Decreased LIF—While Pleiotropic, Expression of Cardioprotection

As a cytokine, LIF regulates embryonic implantation, cell growth, differentiation and survival. LIF is the most pleiotropic interleukin-6 (IL-6), covering a lot of functions in various, if not all, tissues [36,63]. IL-6 is a wide family of cytokines including LIF, IL-6, IL-11, ciliary neurotrophic factor, oncostatin M, cardiotrophin 1, cardiotrophin-like cytokine, and IL-27 [64]. While LIF and IL-6 are part of the same family, they have different effects [36]. LIF is considered one of the most puzzling cytokines by biologists as it is able to induce a very large variety of actions, including platelet formation, bone formation, adipocyte lipid transport, adrenal hormone production, and neuronal formation. With regard to immunity, LIF may contribute to downregulation of inflammation by downregulating the expression of the proinflammatory cytokine tumor necrosis factor (which, however, we did not see in our study) [65]. Considering the Fontan pathophysiology, LIF levels could be related to many systems, i.e., the cardiovascular system, immune system, nervous system, or hematopoietic system. In the heart, LIF protects the myocardium [66,67]. LIF activates JAK-STAT and MAPK in cardiomyocytes for increased survival facing stress [68]. LIF is related to substrate utilization and presumably increases glucose utilization [69]. Chronically activated, this could trigger insulin resistance. LIF relates to cardiac hypertrophy as well as the presence of reactive oxidative species [70]. Pressure overload triggers LIF expression. In a previous study, IL-6 was found to be elevated in stable Fontan patients as a sign of chronic inflammation [71]. There are several reports on a high prevalence of altered glucose metabolism and diabetes mellitus in Fontan patients [16,72,73]. In our study, we found a decreased LIF level in ‘good’ Fontan patients compared to matched controls. This lack of high LIF levels as a protecting factor against hemodynamic or bioenergetic instabilities might suggest that the Fontan heart—even in ‘good’ Fontan patients with a systemic LV—is prone to HF. How LIF levels correlate with IL-6 levels in Fontan patients has not yet been investigated.

LIF is also expressed as a contraction-induced myokine. Theoretically, increased physical activity could produce an increased expression of LIF and regain some cardiac protection. Thus, it would also be interesting to know the LIF levels in the cohort of Tran et al., who, like our group, focused on Fontan patients exhibiting very good exercise capacity (the so-called ‘Super-Fontan’ phenotype) [74]. The cohort of Tran et al. indeed reported high levels of daily physical activity in general, which, unfortunately, we did not inquire about. It would thus be interesting to focus on the actual activity in former studies.

#### 3.2.4. Decreased NGF-β—Alteration of the Autonomic Nervous System, from Cognitive Development to Cardiac Rhythm Abnormalities, and Metalloproteinase Inhibitor

NGF-β is a member of the NGF family of proteins that play a crucial role in the growth, development, maintenance, and survival of nerve cells (neurons). NGF-β is a specific isoform of NGF, and it is involved in various biological processes related to the (autonomous) nervous system, such as cognitive development and heart electrophysiology. NGF-β is found downregulated in several brain diseases, including mild cognitive deficit [75] and ADHD [76]. In neurological conditions, downregulated NGF-β can be associated with increased inflammation [77]. In Fontan patients, neurodevelopmental outcomes and mental health are of major interest, and objectivating the neurodevelopmental and mental health status of our patients would have been helpful in interpreting our finding of decreased NGF-ß levels [78]. Involved in autonomous nervous function, NGF-β is found also impaired in HF and rhythm disturbances [79]. Cardiac ablation of an atrial fibrillation substrate raises NGF-β [80]. While all but 1 out of our 20 patients had normal sinus rhythm and adequate atrioventricular conduction, late Fontan patients are known to suffer overall autonomic dysfunction [45]. A further role of NGF-ß seems to be the inhibition of metalloproteinases [69], and it may be that it promotes tissue remodeling processes such as fibro- and angiogenesis and also thrombosis complications [70].

### 3.3. Combining Four Proteomics-Based Proteins with Traditional Biomarkers for a Global Picture

The third important finding of our study is that in our receiver operating characteristic analysis, with regard to group assignment, the combination of proteomics-based variables and traditional biomarkers performed best in classification (patient vs. control) compared to either solely traditional or solely proteomics variables.

#### 3.3.1. Uric Acid—Antioxidative Scavenger

Uric acid is often solely seen as a waste product of the purine metabolism. However, it bears an important antioxidative scavenger function [81,82]. It also allows us to maintain blood pressure even on low salt and protects the endothelium, erythrocytes, and macrophages [83]. It protects the phospholipid bilayer membrane against peroxidation [82]. Oxidative radicals contribute to the loss of function in the myocardium. Xanthine oxidase (XO) is the key enzyme in uric acid production, expressed in the microvascular endothelium. It is as well a producer as a scavenger of radicals. While producing the antioxidant uric acid, the XO-catalyzed radical formation is held responsible for a large share of the reperfusion damage in the heart, brain, kidney, and intestine [81]. Urate is a co-substrate of cyclooxygenase. The precise role of uric acid in Fontan circulation still needs to be elucidated. Nevertheless, reactive oxygen species are known to be part of chronic inflammation [84], and endogenous uric acid production might be part of a defense mechanism against them. In a previous study, hyperuricemia was predictive of mortality in univariate analysis, but the prognostic value of hyperuricemia might be limited in multivariate analysis [85].

#### 3.3.2. γ-Glutamyl-Transferase (γGT)—Liver Disease, and Antioxidative Glutathione Status

γGT is an enzyme mostly found in the liver and used to assess liver function in fatty liver disease, alcoholism, and NASH syndrome. Less understood is the involvement of γGT in the extracellular catabolism of antioxidative glutathione (maintaining its cellular concentration) and thus its influence on the oxidation-antioxidant balance [86,87]. Liver disease is a major burden for Fontan patients. By adolescence, all of them will have developed silent fibrosis [70]. While elevated γGT can be related to fibrosis, how it relates to the antioxidative status of Fontan patients and if any therapeutic improvement of the oxidation–antioxidant balance could improve the comorbidities seen in this cohort is open to investigation.

#### 3.3.3. Cholesterol—Fontan Dyslipidemia Requiring Further Lipidology Research

Cholesterol is a steroid alcohol essential to life. Most of the quantity available in the human body is of endogenous origin—exogenous cholesterol from nutrition represents but 25% of cholesterol concentration. Cholesterol is necessary as it is an essential building block of the phospholipid bilayer of the cell membrane and a substrate required for steroid hormone synthesis. Previous literature reports on altered cholesterol serum levels in Fontan patients [17,18]—triglycerides, high-density lipoproteins (HDL), and low-density lipoproteins (LDL). Interestingly, only triglycerides and HDL, not non-HDL-cholesterol, differed between groups, and our receiver operating characteristic analysis with regard to patient vs. control showed the high discriminative power of triglycerides and HDL but not of non-HDL-cholesterol. All lipoproteins are necessary for lipid transport as they cover different functions. Apolipoprotein B (LDL) transports triglycerides as a source of energy to the muscle and cholesterol back to the liver/intestine. Apolipoprotein A-I (HDL) transports triglycerides to the tissues producing steroid hormones. The alterations of serum levels of triglycerides, HDL, and reported LDL in Fontan patients might be explained by the consequences of the Fontan circulation itself (elevated central venous pressure, altered liver circulation and mesenteric flow). To confirm this hypothesis, further lipidology studies are required.

### 3.4. Limitations, Confounders

Since Fontan patients are scarce and the cohorts that can be assembled are heterogeneous, in order to limit variability we focused on Fontan patients with a dominant LV who underwent Norwood II and III surgery. Hence, our study group is small. We did not have a chance to replicate our examinations. These points may limit any generalization from our findings.

Also limiting may be the targeted proteomic approach using a commercial kit, as proteins not covered by the kit may be important. However, in terms of project implementation, our choice seemed reasonable. In concert with our metabolomics studies in the same patient (and control) cohort [20,21], the results of our current study and the finding that the proteomics approach is feasible in adult Fontan patients may help in configuring a joint metabolomics and proteomics approach methodologically adapted to requirements for Fontan patients.

We analyzed serum samples, thereby precluding direct comparison between our results and those of studies that used tissue samples. We acknowledge that having assayed serum rather than vascular or myocardial tissue, especially the point of altered myocardial energy metabolism remains speculative.

We cannot rule out certain system-driven aspects of disease pathogenesis; unknown confounders that affect metabolic profiles might be the true basis for the observed differences. The opportunity to correlate our findings with those from invasive assessment would have been advantageous. Furthermore, to distinguish Fontan patients with preserved status from those with impaired hemodynamic status, we should not have focused solely on the configuration of the systemic ventricle, oxygen saturation, exercise capacity, and valve regurgitation; rather, we should have also focused on hemodynamic parameters or the presence of substantial collateral flow. The same is true for liver disease: besides traditional hepatic function/status in the laboratory, we should ideally have included information on liver imaging (incl. elastography). This is part of our current, and larger, approach in progress, part of which is this pilot study. Moreover, differences in body composition or lifestyle may have influenced our results to an unknown degree. We strove to lessen the likelihood of such errors by following a strict inclusion and exclusion protocol, especially with regard to (known) comorbidities, medication, and diet (vegetarian/vegan vs. non-vegetarian/vegan). Further influencing our findings might be the intake of phenprocoumon, which 80% of our patients were taking. As an oral anticoagulant inhibitor of vitamin K oxidoreductase, it interacts with hepatic metabolism and can even induce hepatitis. Socioeconomic status might affect further aspects, like physical activity and diet (beyond being vegetarian/vegan or not). To lessen these confounders, it would have been favorable to include probands from different socioeconomic statuses (in our case, a high percentage of control probands were staff of the university). Lastly, we cannot exclude that differences in body height, weight (BMI), or kidney function between patients and controls confounded our findings; to match probands with controls for those values might be valuable.

## 4. Materials and Methods

### 4.1. Study Design and Inclusion and Exclusion Criteria

This study is a prospective observational cohort study, and it is a sub-work of our main study with the trial registration number: ClinicalTrials.gov Identifier NCT04764305. At the Department of Pediatrics III (Pediatric Cardiology), Medical University of Innsbruck, Austria, and at the Center of Pediatric Cardiology and Congenital Heart Disease, Heart and Diabetes Center North-Rhine Westphalia, Ruhr-University of Bochum, Germany, we prospectively examined a cohort of adult patients after a Fontan procedure (heterogenous regarding history of prior aortic root surgery and of type of surgery) and a second cohort of healthy biventricular controls matched for age and sex. Controls were randomly recruited among staff of the Departments of Pediatrics and Pediatric Cardiology, Medical University of Innsbruck, Austria. Patients were identified through surgical, cardiac catheterization, cMRI, and exercise capacity testing databases. All patients had undergone two-stage palliation with partial and total cavopulmonary anastomosis. None had had aortic reconstruction or aortopulmonary shunting. Patients who had their TCPC at another institution were included in the data set if adequate post-Fontan surveillance was completed at our institution.

Eligibility criteria for all subjects were written informed consent; age at testing of ≥18 years; overnight (at least 8 h) fasting before blood sampling; and morning blood sampling. Additional criteria were for patients with a dominant LV and controls: both a biventricular heart without any structural or functional abnormality and the absence of systemic—including cardiovascular—disease.

Exclusion criteria for both patients and controls were the ingestion of any medication directly affecting the metabolic state (e.g., cholesterol-lowering agents) or hemodynamic state (e.g., beta-blockers, sildenafil), except angiotensin converting enzyme inhibitors, diuretics, and anticoagulants in Fontan patients; atrial or ventricular arrhythmia as assessed by 12-channel electrocardiogram; coronary artery disease (history of myocardial infarction, myocardial revascularization, percutaneous coronary intervention, or coronary artery bypass surgery); failure of the systemic ventricle as assessed by echocardiography; valvular heart disease with stenosis or with worse-than-mild atrioventricular or aortic regurgitation as assessed by echocardiography; recurrent effusions or protein-losing enteropathy; any metabolic disease, such as diabetes mellitus; malignancy or other disease leading to cachexia; liver or renal disease; inflammatory disease such as acute or chronic infection; a myeloproliferative disorder; pregnancy or lactation; multiple organ failure; malnourishment; mental handicap not allowing for valid consent to participate in the study or undertake the precluding treadmill exercise; and uptake of oxygen at the anaerobic threshold (VO_2_AT) <20 mL/kg/min (patients) and <25 mL/kg/min (controls) while testing exercise capacity [12,21].

### 4.2. Routine Examination

Age, sex, weight, body mass index, vital parameters, cardiac risk factors, history of cardiac disease, history and type of cardiovascular surgery, cardiac medication, routine hematological-study biomarkers, and biochemical biomarker profiles were assessed during an outpatient clinic visit. Fasting participants underwent phlebotomy while recumbent, followed by echocardiography using traditional parameters of systolic (LV shortening fraction (SF), ejection fraction (EF)) and diastolic (E/A ratio) function and cavity dimension (Acuson S2000, Siemens Healthineers, Erlangen, Germany).

### 4.3. Proteomics Analysis

#### 4.3.1. Samples and Protein Extraction and Labelling

40 serum samples were examined (20 patients, 20 age- and sex-matched controls). Blood studies required samples 0.5 mL larger than those for routine assessments to allow for determinations of concentrations of protein analytes. However, the affinity-proteomics assay used only 10 µL of sample for the full analysis. After the quality control of the samples, the bulk protein concentration was determined by bicinchoninic acid assay. A reference sample was established by pooling an identical volume of each sample. The samples were labeled at an adjusted protein concentration for two hours with scioDye 2 (Sciomics GmbH, Neckargemünd, Germany). The reference sample was labeled with scioDye 1 (Sciomics GmbH, Neckargemünd, Germany). After two hours, the reaction was stopped, and the buffer was exchanged with PBS. All labeled protein samples were stored at −20 degrees Celsius until use.

#### 4.3.2. Sample Incubation

The 40 samples were analyzed in a dual-color approach using a reference-based design on 40 scioCD antibody microarrays (Sciomics GmbH, Neckargemünd, Germany) targeting different CD surface markers, cytokines, and chemokines. Each antibody is represented on the array in four replicates. The arrays were blocked with scioBlock (Sciomics GmbH, Neckargemünd, Germany) on a Hybstation 4800 (Tecan Austria GmbH, Grödig, Austria). Then, the samples were incubated competitively with the reference sample using a dual-color approach. After incubation for three hours, the slides were thoroughly washed with 1× phosphate-buffered saline with Tween-20 and TritonX-100, rinsed with 0.1× phosphate-buffered saline and water, and subsequently dried with nitrogen.

### 4.4. Data Acquisition and Analysis

Slide scanning was conducted using a Powerscanner (Tecan Austria GmbH, Grödig, Austria) with constant instrument laser power and photomultiplier settings. Spot segmentation was performed with GenePix Pro 6.0 (Molecular Devices, Union City, CA, USA). Acquired raw data were analyzed using the linear models for the microarray data (LIMMA) package of R-Bioconductor after uploading the median signal intensities. For normalization, a specialized invariant Lowess method was applied. For analysis of the samples, a one-factorial linear model was fitted via least-squares regression with LIMMA.

As the data set consisted of matched samples, statistical analysis was performed using a paired *t*-test. All presented *p*-values were adjusted for multiple testing by controlling the false discovery rate according to Benjamini and Hochberg [88]. Proteins were defined as differential (statistically different between patients and controls) for |logFC| > 0.5 and an adjusted *p*-value < 0.05.

Differences in protein abundance between different samples or samples groups are presented as log-fold changes (|logFC|) calculated for the basis 2. In a study comparing samples versus controls, a|logFC| = 1 means that the sample group had, on average, a 2^1^ = 2-fold higher signal than the control group. |logFC| = −1 stands for 2−1 = 1/2 of the signal in the sample compared to the control group.

In addition to the comparison of variable classification impact ranks calculated by classical statistics, we completed classical statistics and random forest RF analyses of the proteomics dataset on the same patient cohort. The model was trained using all available data sets, with parameter settings provided in the legend of Table 4. We used an RF methodology as a predictor selection algorithm for classification and regression (programme RF++ rel. 1.0). Input data for the training model developed were all available data from this proteomics study (526 variables). The output (prediction) was the classification into diseased (Fontan patient) vs. non-diseased (control proband) groups [28].

To evaluate the classification performance (patient vs. control) of the differential proteins in combination with traditional laboratory markers that differed between patients and controls, ROC analysis was applied, and sensitivity, specificity, and AUC were assessed.

Statistical analysis and data visualization were performed using an in-house programmed and optimized data analysis based on R-Bioconductor.

### 4.5. Pathway Analysis

Information on analytes and the pathways in which they are involved is mainly based on https://www.uniprot.org (accessed on 10 December 2023) [27].

## 5. Conclusions

Our study identified four serum proteins, SCD1, GLPA, LIF, and NGF-β, which suggest a distinct pro-inflammatory, pro-fibrotic, and pro-angiogenetic state in the here-examined adult ‘good’ Fontan patients with a systemic LV and good exercise capacity, and without traditional laboratory signs of HF, inflammation, protein loss, or major hepatic function impairment. The classification power with respect to the discrimination of Fontan patients and healthy biventricular controls of the combination of proteomics and traditional variables exceeded that of proteomics variables or traditional biomarkers alone. The here-mentioned proteins might complement traditional diagnostic laboratory and imaging variables, yielding a better understanding of the development of comorbidities in the Fontan circulation. They may play a future role in outcome prediction and the identification of candidate targets for diagnostic and therapeutic approaches.

## Figures and Tables

**Table 1:** “Traditional“ variables by participant group. Major features of the variables of “traditional” examinations (capacity testing, routine laboratory analyses) that differ significantly (student’s *t*-testing, *p* < 0.05) between patients and controls as set out in our related papers on lipid and amino acids metabolism [12,13]. ALT, alanine aminotransferase; AST, aspartate aminotransferase; BP, blood pressure; CRP, C-reactive protein; GLDH, glutamatdehydrogenase; γGT, gamma glutamyl transferase; HDL, high-density lipoprotein; INR, international normalized ratio; NT-proBNP, N-terminal-pro-brain natriuretic peptide; VO_2_AT, oxygen uptake at the anaerobic threshold; VO_2_max, peak oxygen uptake. All values are given as mean ± standard deviation. * NT-pro BNP only of 17 controls available, thus *n* = (17 + 17).

Variable	Unit	Patient	Control	*p*-Value
minimum SpO_2_	%	90 ± 3	98 ± 1	<0.00001
maximum SpO_2_	%	93 ± 3	99 ± 1	<0.00001
VO_2_AT	ml/kg/min	24.5 ± 4.9	30.1 ± 3.6	<0.00001
VO_2_max	ml/kg/min	28.8 ± 10.1	45.7 ± 6.4	<0.00001
heart rate at rest	1/min	83 ± 17	86 ± 19	0.08
BP systolic at rest	mmHg	123 ± 10	119 ± 12	0.04
BP diastolic at rest	mmHg	68 ± 8	71 ± 8	0.04
hematocrit	%	47 ± 5	39 ± 4	<0.00001
hemoglobin	g/dL	16.4 ± 2.1	12.7 ± 1.4	<0.00001
platelet count	1000/nL	171 ± 73	279 ± 88	0.0002
leucocytes	1/nL	6.7 ± 3.2	7.2 ± 2.6	0.23
γGT	U/L	86 ± 43	35 ± 19	0.00002
alkaline phosphatase	U/l	103 ± 53	99 ± 73	0.2
ALT	U/L	39 ± 11	31 ± 10	0.04
AST	U/L	35 ± 8	32 ± 8	0.12
GLDH	U/L	3.7 ± 1.9	3.5 ± 1.5	0.26
total bilirubin	mg/dL	1.22 ± 0.67	0.3 ± 0.29	<0.00001
CRP	mg/dL	0.18 ± 0.2	0.16 ± 0.14	0.47
fibrinogen	mg/dL	239 ± 78	259 ± 57	0.17
antithrombin III	%	101 ± 11	105 ± 9	0.38
INR		2.1 ± 0.7	1 ± 0.04	<0.00001
PTT	sec	35 ± 6	27 ± 4	0.00004
creatine kinase	U/L	129 ± 66	95 ± 38	0.09
uric acid	mg/dL	5.9 ± 1.4	3.9 ± 1.3	0.0003
creatinine	mg/dL	0.8 ± 0.12	0.53 ± 0.18	<0.00001
urea	mg/dL	32 ± 7	23 ± 9	0.0008
triglycerides	mg/dL	128 ± 86	47 ± 22	0.0003
total cholesterol	mg/dL	145 ± 27	149 ± 34	0.77
HDL-cholesterol	mg/dL	42 ± 15	51 ± 22	0.03
non-HDL-cholesterol	mg/dL	85 ± 25	73 ± 21	0.2
total protein	g/dL	7.2 ± 0.5	7.0 ± 0.7	0.31
albumin	mg/dL	4145 ± 492	4215 ± 218	0.64
NT-proBNP *	pg/mL	53 ± 69	41 ± 32	0.41

**Table 2:** Proteins with differential abundance in patient and control samples. Proteins with a positive |logFC| value had a higher abundance in patient samples, and proteins with a negative value had a higher abundance in control samples. In addition, *p*-values adjusted for multiple testing are listed. The Uniprot-Identifier links to the Uniprot-Entry (https://www.uniprot.org [27], accessed on 22 February 2022).

Protein	Antibody ID	Uniprot-Entry-Name	Uniprot ID	logFC	AveExp	adj. *p*-Value	HGNC
SDC1	ab1576	SDC1_HUMAN	P18827	0.96	11.14	2.2 × 10^−2^	SDC1
GLPA	ab1491	GLPA_HUMAN	P02724	0.85	11.09	2.18 × 10^−2^	GYPA
LIF	ab1725	LIF_HUMAN	P15018	−0.6	15.2	5.61 × 10^−8^	LIF
NGF-beta	ab1602	NGF_HUMAN	P01138	−0.75	12.44	6.69 × 10^−3^	NGF

**Table 3:** Other differential proteins. Differential proteins including those that do not reach the |logFC| and significance thresholds simultaneously, i.e., proteins with differential abundance in patient and control samples, which feature either notable or significant |logFC|, while not reaching the significance and |logFC| thresholds simultaneously. Proteins with a positive |logFC| value were abundant in patient samples, and proteins with a negative value in control samples. In addition, *p*-values adjusted for multiple testing are listed. The Uniprot-Identifier links to the Uniprot-Entry (https://www.uniprot.org [27], accessed on 22 February 2022).

Protein	Antibody ID	Uniprot-Entry-Name	Uniprot ID	logFC	AveExp	adj. *p*-Value	HGNC
LYAM3	ab1570	LYAM3_HUMAN	P16109	1.08	11.45	2.17 × 10^−1^	SELP
CD28	ab1420	CD28_HUMAN	P10747	0.82	12.14	2.14 × 10^−1^	CD28
B3GA1	ab1568	B3GA1_HUMAN	Q9P2W7	0.75	11.25	6.28 × 10^−2^	B3GAT1
TNR8	ab1423	TNR8_HUMAN	P28908	0.73	10.92	7.14 × 10^−2^	TNFRSF8
IL18	ab1511	IL18_HUMAN	Q14116	0.69	11.19	2.17 × 10^−1^	IL18
CD33	ab1562	CD33_HUMAN	P20138	0.66	11.31	2.24 × 10^−1^	CD33
ITAE	ab1573	ITAE_HUMAN	P38570	0.66	10.88	1.08 × 10^−1^	ITGAE
IL2RB	ab1575	IL2RB_HUMAN	P14784	0.65	11.18	1.13 × 10^−1^	IL2RB
SLAF1	ab2132	SLAF1_HUMAN	Q13291	0.63	10.35	4.10 × 10^−1^	SLAMF1
FCG2A	ab1561	FCG2A_HUMAN	P12318	0.61	10.85	2.46 × 10^−1^	FCGR2A
PRTN3	ab1501	PRTN3_HUMAN	P24158	0.60	11.04	2.10 × 10^−1^	PRTN3
BDNF	ab1502	BDNF_HUMAN	P23560	0.55	10.96	2.21 × 10^−1^	BDNF
CRLF2	ab1498	CRLF2_HUMAN	Q9HC73	0.53	11.73	2.47 × 10^−1^	CRLF2
ENTP1	ab1111	ENTP1_HUMAN	P49961	0.53	11.36	5.89 × 10^−1^	ENTPD1
VGFR1	ab1618	VGFR1_HUMAN	P17948	0.50	11.63	2.46 × 10^−1^	FLT1
S10A8/9	ab1624			−0.42	14.37	3.94 × 10^−2^	
pan HLA-class II	ab1496			−0.46	9.86	4.53 × 10^−2^	
SDF1	ab2491	SDF1_HUMAN	P48061	−0.46	11.20	5.39 × 10^−3^	CXCL12
TNFB	ab1921	TNFB_HUMAN	P01374	−0.50	12.00	8.04 × 10^−2^	LTA
Lactoferin	ab1611	TRFL_HUMAN	P02788	−0.52	13.03	8.10 × 10^−2^	LTF
PLF4	ab1841	PLF4_HUMAN	P02776	−0.56	12.51	8.61 × 10^−1^	PF4
CD53	ab1453	CD53_HUMAN	P19397	−0.56	13.55	3.50 × 10^−1^	CD53
I17RA	ab2407	I17RA_HUMAN	Q96F46	−0.61	12.62	2.47 × 10^−1^	IL17RA
TNR6	ab1478	TNR6_HUMAN	P25445	−0.66	14.34	1.55 × 10^−1^	FAS

**Table 4:** Random forest analysis and classical statistical analysis of proteomics data (526 proteins); specific serum metabolite and its classification impact compared to classical statistics. Only the topmost 22 random forest ranks are given. In bold are given the topmost ranks of those analytes, which in classical statistics were statistically different between patient and control serum in abundance (adj. *p* < 0.05, these were 7/526). Among the 22/526 topmost RF-ranked analytes we find 3 of those 4 analytes. Random Forest parameter setting: no. of variables used, 526; number of candidate predictors randomly drawn for a split (mtry), 23; no. of trees, 12,000). Random forest: Rank, rank order; score, classification impact score (MDM). Classical statistics: adj., adjusted; fold change, ratio between the patients‘ and the controls‘ serum concentration of metabolites, a positive (negative) value indicates a higher (lower) metabolite concentration in patients; metabolite, name of metabolite; rank, rank order according to hierarchical cluster analysis [28].

Random Forest		Classical Statistics				Protein
Rank	Score	Rank (n = 64)	Fold Change	Adj. *p*	Rank (Only Adj. *p* < 0.05, n = 7)	
1	0.0085	5	−0.75	0.0067	3	NGF-β
2	0.0068	11	−0.6	0.000000056	4	LIF
3	0.0052	28	0.36	0.25		CCL14
4	0.0048	21	0.44	0.22		PD1L1
5	0.0044	25	−0.39	0.22		CCL5
6	0.0041	43	−0.21	0.61		CD38
7	0.0028	19	−0.46	0.0054	6	SDF1
8	0.0024	25	−0.2	0.76		AMPN
9	0.0023	38	−0.26	0.98		LYAM1
10	0.0018	23	0.42	0.25		CADH2
11	0.0017	28	0.36	0.51		VCAM1
12	0.0016	35	0.09	0.74		CD27
13	0.0015	1	1.08	0.98		LYAM3
14	0.0013	41	−0.23	0.59		DPP4
15	0.0011	21	0.1	0.98		CEAM1,3,5,6,8
16	0.001	20	−0.45	0.25		BMP4
17	0.001	30	−0.34	0.55		VGFR2
18	0.001	20	−0.45	0.62		ITA2B (CD41a)
19	0.0009	28	−0.36	0.22		CD9
20	0.0008	35	−0.29	0.54		IL34
21	0.0008	9	0.63	0.41		SLAF1
22	0.0007	2	0.96	0.02	1	SDC1

## Data Availability

The datasets generated and analyzed during the current study are available upon request with appropriate IRB approval.

## Supplementary Materials

The following supporting information can be downloaded at: https://www.mdpi.com/article/10.3390/ijms25105416/s1.
